# Type IV Hiatal Hernia Containing the Gastric Pouch and Proximal Roux Limb: A Rare Cause of Bowel Obstruction Following Roux-en-Y Bypass Surgery

**DOI:** 10.7759/cureus.10132

**Published:** 2020-08-30

**Authors:** Michael E Nance, Emanuel Shapera, Andrew A Wheeler

**Affiliations:** 1 Internal Medicine, University of Missouri-Columbia, Columbia, USA; 2 General Surgery, University of Missouri-Columbia, Columbia, USA; 3 Surgery, University of Missouri-Columbia, Columbia, USA

**Keywords:** roux-en-y gastric bypass, hiatal hernia, small bowel obstruction, weight loss surgery, bariatric surgery, gastric bypass complications, laparoscopic surgery

## Abstract

Roux-en-Y gastric bypass (RYGB) is considered the gold standard for weight loss surgery and is an effective, safe treatment for morbid obesity and associated metabolic derangements. Complications such as small bowel obstruction are rare with a reported incidence of 5%. Obstruction caused by hiatal herniation of the gastric pouch and alimentary limb occurs even less frequently. Prompt recognition and treatment are imperative as delayed intervention may result in significant morbidity. At the time of this manuscript there have only been four reported cases in the literature highlighting a paucity of clinical guidance for the recognition and management of this complication. Here we present a case of acute small bowel obstruction secondary to hiatal herniation of the gastric pouch and proximal Roux limb. Furthermore, we review the literature and discuss the key aspects for the management of this complication.

## Introduction

Roux-en-Y gastric bypass (RYGB) is effective in the treatment of morbid obesity and its associated metabolic derangements [1,2]. However, complications such as small bowel obstruction can rarely occur and may be observed in approximately 5% of cases [3]. Obstruction via hiatal herniation of the gastric pouch and alimentary limb occurs even less frequently, with only four cases reported in the literature [4-7]. We present a case of acute small bowel obstruction 12 years after RYGB caused by herniation of the gastric pouch and proximal Roux limb into a hiatal hernia as well as our management of this condition.

## Case presentation

**Figure 1:**
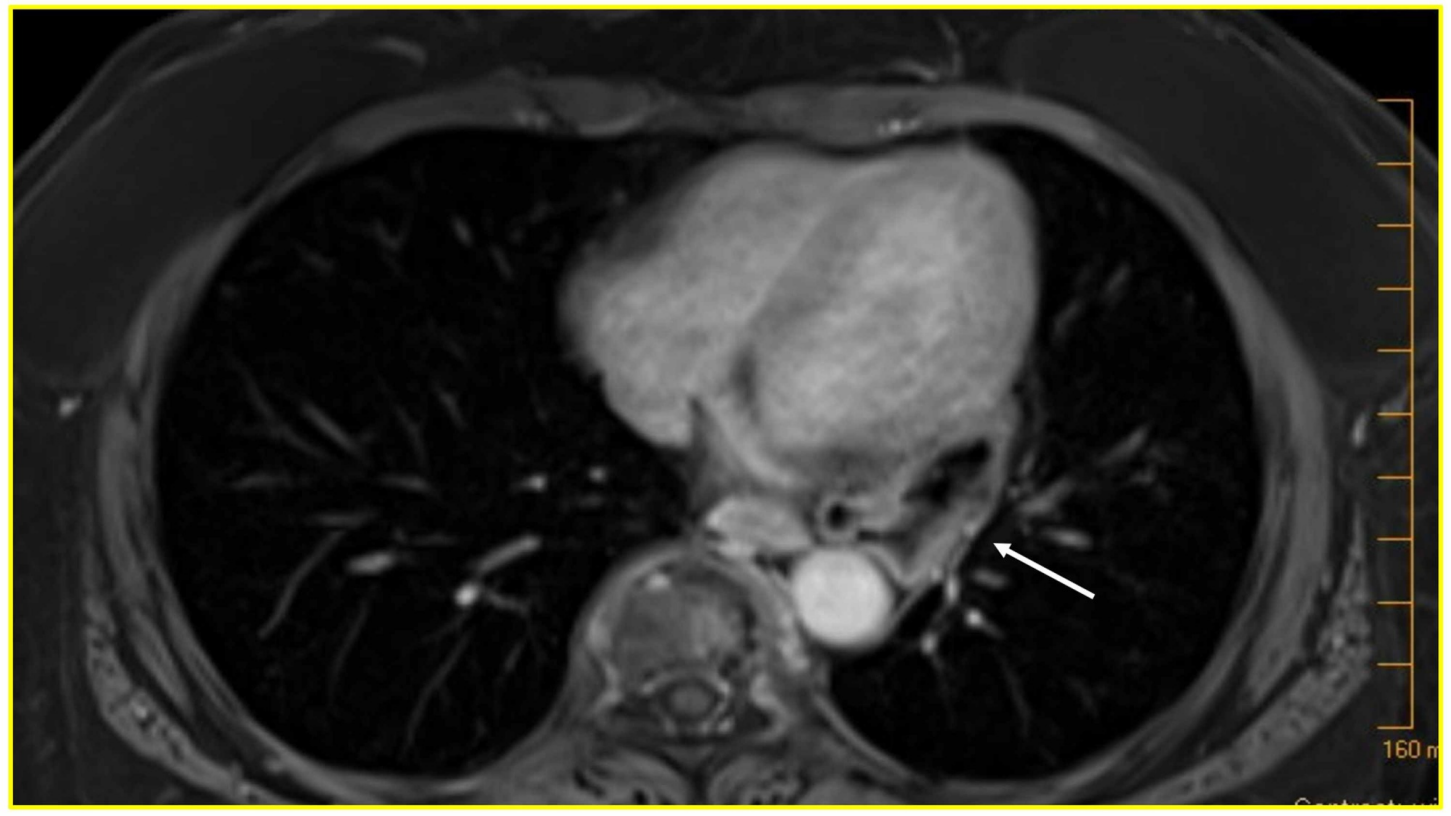
Gastric pouch herniation 12 years post Roux-en-Y bypass surgery T1-weighted axial magnetic resonance image abdomen and pelvis with gadolinium contrast demonstrating the herniated gastric pouch (white arrow, herniated gastric pouch).

**Figure 2:**
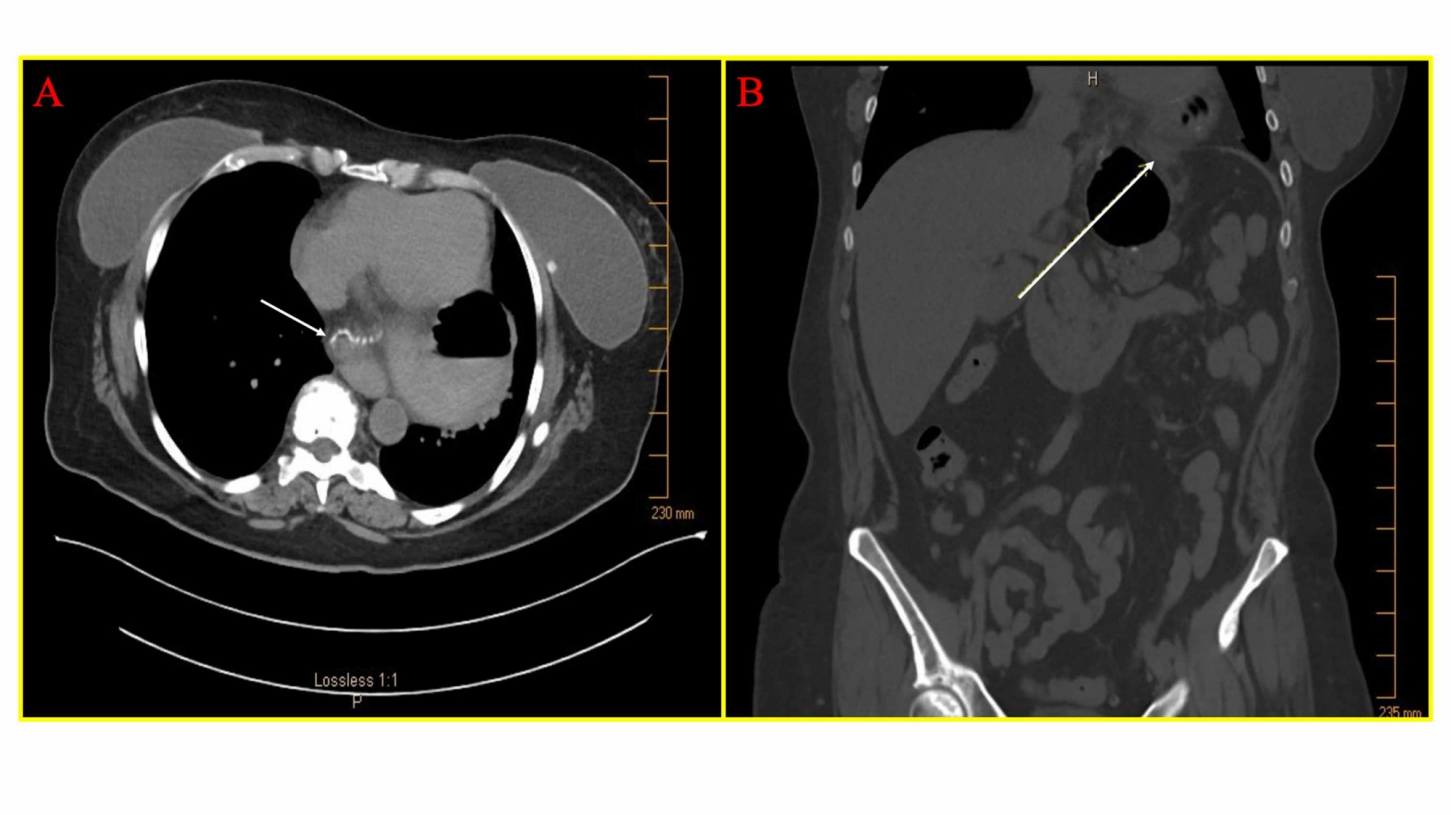
Pre-operative imaging demonstrating gastric pouch and proximal Roux-limb herniation Computed tomography abdomen and pelvis without contrast showing (A) axial and (B) coronal sections demonstrating the herniated Roux limb and gastric pouch prior to surgery (white arrows, herniated Roux limb).

A 68-year-old female presented with a one-year history of progressive nausea, emesis, and sharp epigastric abdominal pain leading to eventual acute intolerance of oral intake for four days. She previously underwent a retro-colic Roux-en-Y gastric bypass (RYGB) surgery 12 years prior for treatment of her morbid obesity. Eight weeks prior to presentation, she underwent magnetic resonance cholangiopancreatography for evaluation of her chronic symptoms. Imaging revealed a large hiatal hernia containing the gastric fundus and the absence of any additional acute processes (Figure 1). On presentation to the emergency department, a computed tomography (CT) scan of abdomen without contrast was obtained due to her worsening epigastric abdominal pain and intolerance to liquids and solids, which showed the gastric pouch and Roux limb herniated into the thorax with obstruction of Roux limb and a clear transition point at the esophageal hiatus (Figure 2A and B). Given these concerning findings, the patient was taken to the operating room for emergent laparoscopic hiatal hernia repair.

An optical trocar was utilized to gain entry into the abdominal cavity. Exploration of the esophageal hiatus was performed. A type IV hiatal hernia involving the entire gastric pouch and proximal Roux limb was identified. The bowel was distended and edematous but viable. The pars flaccida was opened with a laparoscopic bipolar vessel sealer, which demonstrated the left crus being densely adherent to the Roux limb. After careful dissection, the hernia sac and contents including gastric pouch and proximal Roux limb were reduced out of the hiatus. The gastric pouch and Roux limb were closely inspected, both of which appeared viable. The anterior and posterior hernia sacs were separated from the bilateral crus and mediastinal structures until the contents were reduced and adequate esophageal length was obtained. A posterior cruroplasty was performed with 2-0 Ethibond suture. Intraoperative upper endoscopy confirmed smooth passage along the length of the esophagus, a normal appearing gastric pouch, patent gastrojejunostomy free from stricture and ulceration with a dilated yet viable Roux limb. Upper gastrointestinal study on post-operative day one demonstrated no leak or obstruction of contrast passage. She was discharged on post-operative day three with resolution of her symptoms except mild nausea.

At one-, nine-, and 27-month post-operative follow-up visits, the patient reported complete resolution of symptoms, improved tolerance of liquids and solids, and regular bowel function.

## Discussion

According to the American Society for Metabolic and Bariatric Surgery, 252,000 metabolic operations were performed in 2018 for obesity and its associated metabolic derangement, with RYGB accounting for 17% of the total operations [8]. Hiatal hernias are common in patients undergoing weight loss surgery, occurring in up to 40% of patients. Often times, morbidly obese patients have hiatal hernias identified when undergoing weight loss surgery [9]. Utilizing the Metabolic and Bariatric Surgery Association Quality Improvement Project patient use files, paraesophageal hernia repairs have been reported in 16% of patients undergoing weight loss surgery [10]. This represents a significant percentage of weight loss surgery patients who have paraesophageal hernias repaired at the time of their operation, but based on reported rates of hiatal hernias in patients having weight loss surgery this indicates that a significant number of patients do not have their hiatal hernia repaired during their index weight loss surgery. Particularly, small hiatal hernias may not be repaired during the index operation, especially in patients having a gastric bypass compared to sleeve gastrectomy and, thus, may set the patient up for subsequent complications at a remote point from their operation [11].

Hiatal hernia occurrence after RYGB can occur quite frequently after gastric bypass [12]. The relatively small size of the gastric pouch, dissection injury to sling fibers, and tissue strength changes related to rapid weight loss may all predispose patients to post-operative hiatal hernia occurrence after gastric bypass. Several small case series have been published describing the repair of hiatal hernias after gastric bypass with the largest series reporting seven patients with hiatal hernia repair after gastric bypass [9]. In the event of acutely worsening abdominal pain, nausea, and vomiting, clinicians should have a high suspicion for acute small bowel obstruction in RYGB patients and be quick to proceed with evaluation and surgical intervention. Patients with hiatal hernia after gastric bypass may present similar to this patient with chronic symptoms and may include dysphagia, post-prandial epigastric pain, or nausea and/or vomiting [9]. Diagnostic imaging and upper endoscopy may reveal the diagnosis of gastric bypass pouch herniation into the esophageal hiatus, but rarely patients may present acutely with a bowel obstruction from Roux limb herniation.

A review of the literature identified four reported cases making our patient only the fifth reported case of bowel obstruction from a hiatal hernia after gastric bypass (Table 1) [4-7]. Of the four previous cases, all presented with abdominal pain, nausea, and emesis. Additional reported symptoms included shortness of breath and constipation. Time to presentation ranged from four to 48 months (mean 16.75 months) after the index RYGB. Only one case included herniation of the omentum and transverse colon in addition to the gastric pouch and Roux limb. Two of four cases (50%) had a history of hiatal hernia repair at the time of RYGB, thus representing a recurrent hiatal hernia. All cases were successfully managed with anterior (1/4 cases) or posterior (3/4 cases) cruroplasty with or without mesh and did not require bowel resection. Follow-up ranged from four weeks to 12 months with no reported recurrence of symptoms. Due to the limited number of cases, it was not possible to evaluate any effect of hiatal hernia repair at the time of RYGB, gastric pouch size, amount of weight loss, or retro-colic versus ante-colic Roux limb position.

**Table 1:** Cases of Roux limb hiatal hernia causing bowel obstruction

Case #	Operative diagnoses	Sex	Age	Presentation	Weight loss	Time post-RYGB	HH repair	Bowel viable	Symptom recurrence	Comments	Reference
1	Hiatal hernia containing gastric pouch, Roux limb, omentum, and transverse colon	F	43	Constant upper abdominal pain with vomiting, anorexia, shortness of breath, and constipation	>82% excess weight	9 months	Laparoscopic; cruroplasty with mesh	Yes	None; 4-week follow-up	No hernia sac indicating acute herniation without trauma	Borg et al. 2011 [5]
2	Hiatal hernia containing gastric pouch and entire Roux limb	F	47	Sudden onset epigastric pain with nausea and vomiting	71 pounds	6 months	Laparoscopic; posterior cruroplasty with mesh	Yes	None; 6-month follow-up	HH repair at time of RYGB	Caceres et al. 2010 [6]
3	Hiatal hernia containing gastric pouch and proximal Roux limb	F	28	Severe, colicky epigastric pain with nausea and vomiting	Not reported	4 months	Open; anterior cruroplasty without mesh	Yes	None; 12-month follow-up	HH repair at time of RYGB	Cardaci et al. 2017 [7]
4	Hiatal hernia containing gastric pouch and proximal Roux limb	F	68	Sudden onset mid-epigastric pain	150 pounds	48 months	Laparoscopic; cruroplasty without mesh	Yes	Not reported	None	Pandya et al. 2011 [4]
Notes: RYGB, Roux-en-Y-gastric bypass; HH, hiatal hernia.											

In the presented case, a diagnosis of obstruction was suspected based on the patient’s history of protracted, intermittent abdominal pain, nausea, vomiting, and constipation. Although a large hiatal hernia containing gastric pouch was identified on MRI, the Roux limb was not within the hernia, suggesting spontaneous herniation of the alimentary limb leading to the patient’s acute-on-chronic symptoms. This would explain the patient’s prior intermittent symptoms, which worsened upon Roux limb incarceration a few days prior to presentation. This phenomenon of acute-on-chronic symptoms has also been observed with internal hernias leading to acute presentation requiring operative intervention [13,14].

A laparoscopic approach to this patient was undertaken for the demonstrated benefits of laparoscopic versus open paraesophageal hernia repair in the non-bariatric surgery patient [15]. Key tenets of the repair of hiatal hernias after gastric bypass include those pertinent to non-gastric bypass patients with what we feel are some additional components when repairing hiatal hernias after gastric bypass [16]. First, it is important to carefully dissect the gastric pouch along its lateral margin where it is often densely adherent to the remnant stomach due to the adjacent staple lines. At this point, separation of the gastric pouch and remnant stomach should be undertaken if any cephalad retraction of the pouch is present once the rest of the hernia reduced, if adequate intra-abdominal esophageal length cannot be obtained, or to aid in adequate exposure of the left diaphragmatic crus. Next, care should be undertaken along the left crura and left side of mediastinum as the gastric pouch staple line can be densely adherent to adjacent structures. Bioabsorbable mesh can be considered depending on the status of the cruroplasty. Lastly, internal hernias should be inspected for during the operation as well as anytime a patient with a gastric bypass is returned to the operating room for an intra-abdominal procedure.

Small bowel obstruction after RYGB often requires urgent surgical intervention as compared to small bowel obstruction in non-bariatric patients where a period of non-operative therapy can be trialed. Therefore, timely diagnosis is critical to provide timely operative intervention. In the absence of more common causes of bowel obstruction after RYGB, such as internal hernia, abdominal adhesions, or even small bowel intussusception, the treating physician must be aware of the possibility of hiatal herniation of the alimentary limb as a possible cause to avoid a delay in diagnosis and treatment.

## Conclusions

Bowel obstruction after RYGB is not uncommon. Obstruction from a hiatal hernia containing the gastric pouch and Roux limb is infrequent but carries the potential for significant morbidity. Treating physicians must remain cognizant of this possibility as prompt diagnosis and treatment are crucial. Laparoscopic repair is safe, effective, and can permit fast recovery. This case report demonstrates an appropriate course of management for patients to consider when treating this condition.
